# Sociodemographic characteristics and frequency of consuming home-cooked meals and meals from out-of-home sources: cross-sectional analysis of a population-based cohort study

**DOI:** 10.1017/S1368980018000812

**Published:** 2018-04-11

**Authors:** Susanna Mills, Jean Adams, Wendy Wrieden, Martin White, Heather Brown

**Affiliations:** 1 Institute of Health & Society, Newcastle University, Baddiley-Clark Building, Richardson Road, Newcastle upon Tyne NE2 4AX, UK; 2 Centre for Diet and Activity Research (CEDAR), MRC Epidemiology Unit, University of Cambridge School of Clinical Medicine, Cambridge, UK; 3 Human Nutrition Research Centre, Institute of Health & Society, Newcastle University, Newcastle upon Tyne, UK

**Keywords:** Home cooking, Diet, Sociodemographic characteristics, UK

## Abstract

**Objective:**

To identify sociodemographic characteristics associated with frequency of consuming home-cooked meals and meals from out-of-home sources.

**Design:**

Cross-sectional analysis of a population-based cohort study. Frequency of consuming home-cooked meals, ready meals, takeaways and meals out were derived from a participant questionnaire. Sociodemographic characteristics regarding sex, age, ethnicity, working overtime and socio-economic status (SES; measured by household income, educational attainment, occupational status and employment status) were self-reported. Sociodemographic differences in higher *v.* lower meal consumption frequency were explored using logistic regression, adjusted for other key sociodemographic variables.

**Setting:**

Cambridgeshire, UK.

**Subjects:**

Fenland Study participants (*n* 11 326), aged 29–64 years at baseline.

**Results:**

Eating home-cooked meals more frequently was associated with being female, older, of higher SES (measured by greater educational attainment and household income) and not working overtime. Being male was associated with a higher frequency of consumption for all out-of-home meal types. Consuming takeaways more frequently was associated with lower SES (measured by lower educational attainment and household income), whereas eating out more frequently was associated with higher SES (measured by greater educational attainment and household income) and working overtime.

**Conclusions:**

Sociodemographic characteristics associated with frequency of eating meals from different out-of-home sources varied according to meal source. Findings may be used to target public health policies and interventions for promoting healthier diets and dietary-related health towards people consuming home-cooked meals less frequently, such as men, those with lower educational attainment and household income, and overtime workers.

Convenience foods, including ready meals, takeaways, fast food and meals from restaurants, have been linked with obesity and diet-related non-communicable diseases^(^ ^1^ ^–^ ^6^ ^)^. Patterns of meal consumption and sourcing have changed in the majority of developed countries since the mid-20th century, with a decline in cooking at home from basic ingredients^(^ ^7^ ^–^ ^9^ ^)^. Adults in the UK still spend approximately three-quarters of their weekly food and non-alcoholic drink budget on eating at home^(^ ^10^ ^)^, although the nature of these meals and degree of involvement in their preparation are not clearly specified. Changes in meal habits and food spending towards an increased reliance on meals from out-of-home sources have been blamed for increases in the prevalence of diet-related non-communicable diseases and obesity^(^ ^11^ ^)^.

In developed countries, cooking (including skills, frequency and extent of involvement) and frequency of eating meals prepared at home have been associated with a range of advantages. These include dietary benefits, such as increased intake of certain nutrients (for example calcium)^(^ ^12^ ^)^, decreased intake of certain nutrients (for example fat)^(^ ^13^ ^)^, consumption of healthier food groups^(^ ^14^ ^–^ ^16^ ^)^ and adherence to dietary guidelines^(^ ^12^ ^)^, and health gains, such as longer lifespan and decreased risk of chronic diseases^(^ ^17^ ^–^ ^19^ ^)^.

In contrast, other research has suggested that supermarket-brand ready meals may be healthier than popular recipes for preparing home-cooked meals^(^ ^20^ ^)^ and that celebrity chef recipes for meals prepared at home may be of poor nutritional quality^(^ ^21^ ^)^. However, these studies used recipes as a proxy for actual meal content^(^ ^20^ ^,^ ^21^ ^)^. Increased time spent in food preparation and clean-up has been associated with elevated cardiometabolic risks among certain population groups, such as middle-aged women^(^ ^22^ ^)^. A higher frequency of meals prepared at home by a caregiver^(^ ^23^ ^)^ and watching cooking shows and cooking from scratch frequently^(^ ^24^ ^)^ have been linked with increased BMI. Some of this complexity may be attributable to geographical variation between studies, and hence cultural differences in food preparation, as well as poorly defined terminology around both home-cooked meals and main meal alternatives. A previous systematic review identified that ‘eating out of home’ may be used to describe food consumed at home but prepared away from home, food prepared at home but consumed away from home, and food both eaten and prepared away from home^(^ ^25^ ^)^. However, despite these inconsistencies in the evidence base, meal source appears to be an important determinant of diet and health.

In an effort to improve population diets and diet-related non-communicable diseases, public health interventions have been developed to preferentially encourage different patterns of meal sourcing, such as through improving cooking skills^(^ ^26^ ^,^ ^27^ ^)^. However, few studies to date have specifically identified the sociodemographic characteristics of those currently engaging in different meal sourcing patterns, which is important to inform targeting and tailoring of public health interventions. In terms of cooking at home, positive associations have been identified with being female, married, older, having dependants at home and greater time availability, whereas the relationship between socio-economic status (SES) and cooking is inconsistent, even in studies using the same socio-economic indicators^(^ ^28^ ^,^ ^29^ ^)^. Studies have linked greater involvement in cooking at home among young adults with higher SES, as measured by educational attainment and occupational status^(^ ^14^ ^)^. In contrast, greater involvement in home food preparation has been associated with lower SES, in terms of household income, among men^(^ ^30^ ^)^ and parental educational attainment, among adolescents^(^ ^31^ ^)^. Involvement in cooking at home has also shown an equivocal relationship with SES, as measured by parental educational attainment, among young adults^(^ ^12^ ^)^ and educational attainment and household wealth^(^ ^32^ ^)^.

Eating meals out more frequently has been associated with being younger and living in a higher-SES household^(^ ^33^ ^)^. In contrast, there is little evidence for a gradient in adult fast-food intake with regard to income and wealth^(^ ^34^ ^)^. Working patterns may be associated with patterns of meal sourcing, given that particularly among employed parents, those with longer working hours or erratic schedules may be more likely to opt for alternatives to home-cooked meals due to time pressures^(^ ^35^ ^)^.

In view of the current mixed picture regarding relationships between consumption of different meal types and varied demographic factors and indicators of SES, further clarity is required. Different measures of SES are likely to reflect different facets of this influence on behaviour, which will in turn help to inform targeting of public health interventions encouraging healthier eating practices. In the present study we aimed to identify detailed sociodemographic characteristics associated with frequency of consuming home-cooked meals and meals from different out-of-home sources, namely takeaways, pre-prepared ready meals and eating out, in a population-based cross-sectional cohort.

## Methods

### Data source

The Fenland Study is a large, population-based cohort study which recruited adults from general practice lists in Cambridgeshire, UK, between 2005 and 2015, inviting those who were born between 1950 and 1975^(^ ^36^ ^–^ ^39^ ^)^. A total of 12 434 participants undertook a comprehensive range of clinical measurements and completed a questionnaire including items on meal patterns and a range of sociodemographic variables. The data collection tools are available online^(^ ^40^ ^)^.

The Fenland Study was approved by the East of England Cambridge Central Health Research Authority National Research Ethics Service Committee and performed in accordance with the Declaration of Helsinki. All participants provided written informed consent to take part. Exclusion criteria for the study included terminal illness, psychosis, pregnancy, inability to walk unaided and previously diagnosed diabetes. The present study has been reported according to the STROBE-nut (Strengthening the Reporting of Observational Studies in Epidemiology–Nutritional Epidemiology) guidelines^(^ ^41^ ^)^.

### Frequency of consuming main meals from different sources

Participants were dichotomised based on their consumption of the main meal of the day from four different sources. Items in the participant questionnaire were: ‘When eating your main meal at home, how often do you usually eat home-cooked meals?’, ‘When eating your main meal at home, how often do you usually eat home delivery or takeaway meals?’ and ‘When eating your main meal at home, how often do you usually eat ready-made meals/prepared foods?’ The main meal was not further defined and interpretation was therefore reliant on each participant. Response options for each question were: ‘never or rarely’, ‘one to two times per week’, ‘three to five times per week’ or ‘more than five times per week’. To ensure comparability between the different main meal sources, consumption frequency was collapsed into ‘two times per week or less’ and ‘more than two times per week’. This accounted for the unequal distribution of consumption frequencies and ensured sufficient numbers in each category to give adequate power for statistical analysis.

Frequency of eating out was established through a separate item in the participant questionnaire: ‘On average, how often do you eat a meal outside of the home (restaurants, pubs, fast-food outlets etc.)?’ Response options were: ‘less than once a week’, ‘once a week’, ‘two to four times a week’, ‘five to six times a week’, ‘once a day’ or ‘more than once a day’. These options were collapsed into ‘less than once per week’ and ‘once or more per week’, for the same reasons as the other main meal sources.

### Sociodemographic characteristics

In view of current evidence regarding factors influencing dietary intake^(^ ^42^ ^)^, we explored patterns of meal consumption according to the following sociodemographic variables: sex, age, ethnicity, working overtime, and SES in terms of household income, educational attainment, occupational status and employment status. Ethnicity was collapsed from the seventeen categories of the 2001 UK Census class^(^ ^43^ ^)^ into white and non-white groups, given the very low prevalence of ethnic minorities. Participants were asked whether they had been employed in the past four weeks and those responding positively were classified as currently working. Participants working more than 48 h in any one of the last four weeks were classified as working overtime, and those who were not currently working were automatically allocated a ‘no’ status in analyses for overtime working. Current or most recent occupation was collapsed into the three hierarchical strata of ‘higher managerial, administrative and professional occupations’ (higher), ‘intermediate occupations’ (middle) and ‘routine and manual occupations’ (routine), according to the National Statistics Socio-economic classification (NS-SEC)^(^ ^44^ ^)^. Annual household income was divided into three categories in the questionnaire: ‘less than £20 000’, ‘£20 000–40 000’ and ‘more than £40 000’. Information on household composition was not available to equivalise household income. Educational qualifications attained were stratified into: ‘no or compulsory school-level qualifications’ (basic), ‘university entry qualifications and vocational equivalents’ (further) and ‘degree level qualifications’ (degree level). Age was considered in single year increments.

### Analytical approach

Participants with missing data on any of the variables described were excluded from the analyses, and differences between included and excluded populations were explored using the Mann–Whitney test with *z* score for continuous variables and the Pearson *χ* ^2^ test for categorical variables. A complete case analysis was performed and unadjusted differences in the frequency of consuming home-cooked meals, takeaways, ready meals and meals out were compared for each sociodemographic characteristic using binary logistic regression. The lower consumption frequency category was used as the reference category. Models were then each adjusted for all sociodemographic variables included, except the specific sociodemographic variable under study. For example, when examining the association between frequency of consuming home-cooked meals and sex, the model was adjusted for all sociodemographic variables except sex. Sociodemographic variables were chosen due to their known or likely confounding relationship with food preparation behaviour. All analyses were conducted using the statistical software package Stata version 14. In view of the large number of comparisons, 99 % CI were used and *P*<0·01 taken to indicate statistical significance.

## Results

Of 12 434 baseline participants in the Fenland Study, full data were available for 11 326 (91·1 %), who were included in analyses. The majority of excluded participants were excluded due to missing data on occupational status or ethnicity. A comparison of participant characteristics for those included and excluded from the analytic sample is shown in Table 1. Included and excluded populations differed significantly in terms of all characteristics (sex, age, ethnicity, annual household income, educational attainment, employment status, working overtime, occupational status and consumption of home-cooked meals and ready meals), except for consumption of takeaways and meals eaten out.

**Table 1:** Characteristics of participants included and excluded from the analytic sample: adults (*n* 11 326) aged 29–64 years at baseline (recruited between 2005 and 2015), Fenland Study, Cambridge, UK

		Included		Excluded		
Variable*	Level	*n*	%	*n*	%	Statistical test (df) and *P* value†
All	–	11 326	91·09	1108	8·91	–
Sex	Male	5291	46·72	452	40·79	*χ* ^2^(1)=14·24, *P*<0·001
	Female	6035	53·28	656	59·21	
Age (years)						
Median	–	48.9		47·1		*z*=−8·17, *P*<0·001
IQR	–	42·9–54·8		40·2–53·4		
Ethnicity	Non-white	305	2·69	34	6·81	*χ* ^2^(1)=29·14, *P*<0·001
	White	11 021	97·31	465	93·19	
Annual household income (£)	<20 000	1529	13·50	139	18·34	*χ* ^2^(2)=30·18, *P*<0·001
	20 000–40 000	3971	35·06	303	39·97	
	>40 000	5826	51·44	316	41·69	
Educational qualifications	Basic	2149	18·97	310	30·21	*χ* ^2^(2)=104·18, *P*<0·001
	Further	5222	46·11	487	47·47	
	Degree level	3955	34·92	229	22·32	
Working in past 4 weeks	No	1398	12·34	778	70·22	*χ* ^2^(1)=2·3×10^3^, *P*<0·001
	Yes	9928	87·66	330	29·78	
Overtime work (>48 h/week)	No	9975	88·07	1037	96·92	*χ* ^2^(1)=77·10, *P*<0·001
	Yes	1351	11·93	33	3·08	
Occupational status	Routine	1814	16·02	158	37·44	*χ* ^2^(2)=168·32, *P*<0·001
	Middle	3372	29·77	147	34·83	
	Higher	6140	54·21	117	27·73	
Home-cooked meals	≤2 times/week	692	6·11	91	8·39	*χ* ^2^(1)=8·74, *P*=0·003
	>2 times/week	10 634	93·89	993	91·61	
Ready meals	≤2 times/week	10 692	94·40	981	92·46	*χ* ^2^(1)=6·74, *P*=0·009
	>2 times/week	634	5·60	80	7·54	
Takeaways	≤2 times/week	10 609	93·67	982	92·38	*χ* ^2^(1)=2·68, *P*=0·102
	>2 times/week	717	6·33	81	7·62	
Eating out	<1 time/week	7695	67·94	764	70·54	*χ* ^2^(1)=3·09, *P*=0·079
	≥1 time/week	3631	32·06	319	29·46	

* Results shown as number and column percentage for categorical variables; median and interquartile range (IQR) for continuous variables (age).

† Test for significant differences between included and excluded populations using the Pearson *χ* ^2^ test for categorical variables; the Mann-Whitney test with *z* score for continuous variables (age).

Slightly over half of participants included were female (53·3 %), most were of white ethnicity (97·3 %) and median age was 48·9 years. The majority of included participants were working (87·7 %) and did not work overtime (88·1 %). Most of the included sample lived in a household with annual income of at least £20 000 (86·5 %). The majority of participants had educational qualifications below degree level (65·1 %) and were in the higher occupational status group (54·2 %). With regard to main meal consumption, most participants ate home-cooked meals as their main meal at home more than twice per week (93·9 %). In contrast, the majority of participants ate ready meals (94·4 %) and takeaways (93·7 %) only twice per week or less. Most included participants ate out less than once per week (67·9 %).

**Fig. 1:**
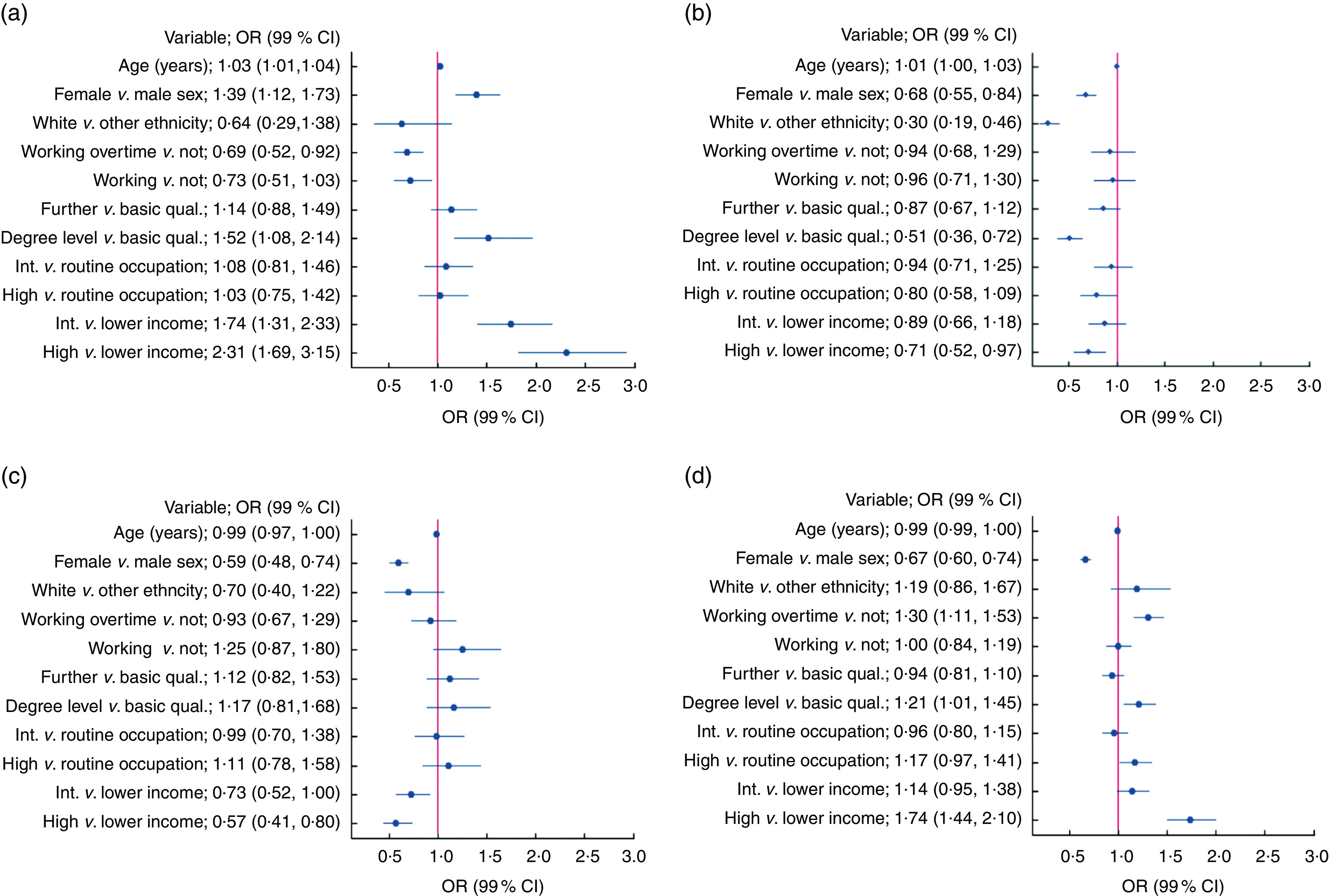
(colour online) Adjusted logistic regressions for associations between sociodemographic characteristics and frequency of consuming home-cooked meals and meals from out-of-home sources among adults (*n* 11 326) aged 29–64 years at baseline (recruited between 2005 and 2015), Fenland Study, Cambridge, UK. OR (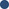) and 99 % CI (represented by horizontal lines) for the frequency of consuming: (a) home-cooked meals >2 *v*. ≤2 times/week; (b) takeaways >2 *v*. ≤2 times/week; (c) ready meals >2 *v*. ≤2 times/week; and (d) eating out ≥1 *v*. <1 time/week. Logistic regressions mutually adjusted, as appropriate, for sex, age, ethnicity, educational attainment, occupational status, household income, employment status and working overtime (int., intermediate; qual., qualifications)

Table 2 shows descriptive statistics for participant sociodemographic characteristics against frequency of consuming home-cooked meals, ready meals, takeaways and meals out. Unadjusted associations are summarised in Table 3 and mutually adjusted associations are presented in Fig. 1.

**Table 2:** Characteristics of participants overall and by frequency of consuming different main meal types: adults (*n* 11 326) aged 29–64 years at baseline (recruited between 2005 and 2015), Fenland Study, Cambridge, UK

			Home-cooked meals				Ready meals				Takeaways				Eating out			
		Total	≤2 times/week		>2 times/week		≤2 times/week		>2 times/week		≤2 times/week		>2 times/week		<1 time/week		≥1 time/week	
Variable*	Level	(*n*11 326)	*n*	%	*n*	%	*n*	%	*n*	%	*n*	%	*n*	%	*n*	%	*n*	%
Sex	Male	5291	377	7·13	4914	92·87	4923	93·04	368	6·96	4903	92·67	388	7·33	3291	62·20	2000	37·80
	Female	6035	315	5·22	5720	94·78	5769	95·59	266	4·41	5706	94·55	329	5·45	4404	72·97	1631	27·03
Age (years)																		
Median	–	48·9	47·2		49·1		49·0		48·2		48·9		49·8		49·2		48·5	
IQR	–	42·9–54·8	41·9–53·3		43·0–54·9		43·0–54·9		42·0–54·0		42·9–54·8		43·5–55·6		43·1–55·0		42·3–54.6	
Ethnicity	Non-white	305	12	3·93	293	96·07	280	91·80	25	8·20	261	85·57	44	14·43	212	69·51	93	30·49
	White	11 021	680	6·17	10 341	93·83	10 412	94·47	609	5·53	10 348	93·89	673	6·11	7483	67·90	3538	32·10
Annual household income (£)	<20 000	1529	151	9·88	1378	90·12	1423	93·07	106	6·93	1396	91·30	133	8·70	1189	77·76	340	22·24
	20 000–40 000	3971	258	6·50	3713	93·50	3742	94·23	229	5·77	3673	92·50	298	7·50	2936	73·94	1035	26·06
	>40 000	5826	283	4·86	5543	95·14	5527	94·87	299	5·13	5540	95·09	286	4·91	3570	61·28	2256	38·72
Qualifications	Basic	2149	170	7·91	1979	92·09	2039	94·88	110	5·12	1959	91·16	190	8·84	1569	73·01	580	26·99
	Further	5222	342	6·55	4880	93·45	4928	94·37	294	5·63	4851	92·90	371	7·10	3714	71·12	1508	28·88
	Degree level	3955	180	4·55	3775	95·45	3725	94·18	230	5·82	3799	96·06	156	3·94	2412	60·99	1543	39·01
Occupation status	Routine	1814	152	8·38	1662	91·62	1709	94·21	105	5·79	1650	90·96	164	9·04	1343	74·04	471	25·96
	Middle	3372	214	6·35	3158	93·65	3195	94·75	177	5·25	3120	92·53	252	7·47	2470	73·25	902	26·75
	Higher	6140	326	5·31	5814	94·69	5788	94·27	352	5·73	5839	95·10	301	4·90	3882	63·22	2258	36·78
Working in past 4 weeks	No	1398	68	4·86	1330	95·14	1334	95·42	64	4·58	1298	92·85	100	7·15	1016	72·68	382	27·32
	Yes	9928	624	6·29	9304	93·71	9358	94·26	570	5·74	9311	93·79	617	6·21	6679	67·27	3249	32·73
Overtime working (>48 h/week)	No	9975	574	5·75	9401	94·25	9422	94·46	553	5·54	9345	93·68	630	6·32	6904	69·21	3071	30·79
	Yes	1351	118	8·73	1233	91·27	1270	94·00	81	6·00	1264	93·56	87	6·44	791	58·55	560	41·45

* Results shown as number and row percentage for categorical variables; median and interquartile range (IQR) for continuous variables (age).

**Table 3:** Unadjusted logistic regressions of associations between the frequency of consuming main meal types and sociodemographic characteristics among adults (*n* 11 326) aged 29–64 years at baseline (recruited between 2005 and 2015), Fenland Study, Cambridge, UK

	Unadjusted odds consuming main meal type more *v.* less frequently							
	Home-cooked meals (>2 *v.* ≤2 times/week)		Ready meals (>2 *v.* ≤2 times/week)		Takeaways (>2 *v.* ≤2 times/week)		Eating out (≥1 *v.* <1 time/week)	
Variable	OR	99 % CI	OR	99 % CI	OR	99 % CI	OR	99 % CI
Sex (female *v.* male)	1·39	1·14, 1·71	0·62	0·50, 0·76	0·73	0·60, 0·89	0·61	0·55, 0·68
Age (years)	1·02	1·01, 1·04	0·99	0·97, 1·00	1·02	1·00, 1·03	0·99	0·98, 1·00
Ethnicity (white *v.* non-white)	0·62	0·29, 1·34	0·66	0·38, 1·13	0·39	0·25, 0·59	1·08	0·78, 1·49
Qualifications (further *v.* basic)	1·23	0·95, 1·58	1·11	0·82, 1·49	0·79	0·62, 1·00	1·10	0·95, 1·27
Qualifications (degree level *v.* basic)	1·80	1·36, 2·39	1·14	0·84, 1·56	0·42	0·32, 0·56	1·73	1·49, 2·01
Occupation (intermediate *v.* routine)	1·35	1·02, 1·79	0·90	0·65, 1·25	0·81	0·62, 1·06	1·04	0·88, 1·23
Occupation (higher *v.* routine)	1·63	1·25, 2·12	0·99	0·74, 1·33	0·52	0·40, 0·67	1·66	1·42, 1·93
Income (£20 000–40 000 *v.* <£20 000)	1·58	1·20, 2·08	0·82	0·60, 1·12	0·85	0·64, 1·13	1·23	1·03, 1·48
Income (>£40 000 *v.* <£20 000)	2·15	1·64, 2·81	0·73	0·54, 0·98	0·54	0·41, 0·72	2·21	1·86, 2·63
Working (in work *v.* not)	0·76	0·54, 1·07	1·27	0·90, 1·80	0·86	0·64, 1·15	1·29	1·10, 1·52
Overtime (work >48 h/week *v.* not)	0·64	0·49, 0·84	1·09	0·79, 1·49	1·02	0·75, 1·38	1·59	1·37, 1·85

As shown in Fig 1, higher odds of eating home-cooked meals more than twice per week was associated with being female (OR=1·39; 99 % CI 1·12, 1·73), whereas being female was associated with lower odds of consuming all out-of-home main meal types more frequently. We found a small association between older age and eating home-cooked meals more frequently (OR=1·03; 99 % CI 1·01, 1·04), although older people did not eat meals from out-of-home sources less frequently than younger people.

Relationships between SES and meal consumption were not consistent across all measures of SES. There was no association between employment status and meal consumption frequency for any type of meal. Higher odds of eating home-cooked meals more than twice per week was associated with higher educational attainment (degree level *v*. basic: OR=1·52; 99 % CI 1·08, 2·14) and greater household income (>£40 000 *v*. <£20 000: OR=2·31; 99 % CI 1·69, 3·15), although the association with higher occupational status was no longer significant after adjustment for other sociodemographic variables. Higher odds of eating out more than once per week was associated with having degree level, compared with basic, educational qualifications (OR=1·21; 99 % CI 1·01, 1·45) and household income above £40 000, compared with below £20 000 (OR=1·74; 99 % CI 1·44, 2·10). The associations between eating out and higher occupational status, and employment status, were no longer significant after adjustment for other sociodemographic variables. Lower odds of eating ready meals more than twice per week was associated with household income above £40 000, compared with below £20 000 (OR=0·57; 99 % CI 0·41, 0·80). Lower odds of eating takeaways more than twice per week was associated with having degree level, compared with basic, educational qualifications (OR=0·51; 99 % CI 0·36, 0·72), and household income above £40 000, compared with below £20 000 (OR=0·71; 99 % CI 0·52, 0·97), although the association with higher occupational status was no longer significant after adjustment for other sociodemographic variables.

White ethnicity was associated with lower odds of eating takeaways more than twice per week (OR=0·30; 99 % CI 0·19, 0·46), although there were no other associations between ethnicity and meal consumption frequency for any other meal type. Working overtime was associated with lower odds of eating home-cooked meals more than twice per week (OR=0·69; 99 % CI 0·52, 0·92) and higher odds of eating out once or more per week (OR=1·30; 99 % CI 1·11, 1·53).

## Discussion

### Statement of principal findings

To our knowledge, the present study is the first large-scale, population-based analysis to describe and compare the sociodemographic characteristics of people consuming home-cooked main meals and meals from different out-of-home sources. These findings should be important in guiding the targeting of public health policies to promote healthier eating patterns and tailoring of associated interventions.

The majority of participants (93·9 %) ate home-cooked meals as their main meal at home more than twice per week, whereas few ate ready meals (5·6 %) or takeaways (6·3 %) more than twice per week. Most participants ate out less than once per week (67·9 %). In fully adjusted analyses, consuming home-cooked meals more frequently was associated with being female, older, not working overtime and higher SES, as measured by greater educational attainment and household income. Eating ready meals more frequently was associated with lower SES in terms of income, and eating takeaways more frequently was associated with lower SES in terms of both income and educational attainment. A higher frequency of eating meals out was associated with being male, working overtime, and higher SES in terms of greater income and educational attainment. Being female was associated with a lower frequency of consuming each of the main meal types from out-of-home sources and a higher frequency of consuming home-cooked meals.

### Strengths and weaknesses of the study

This research used data from the Fenland Study, which is a large population-based cohort study. Participants were drawn from the English county of Cambridgeshire, which is representative of the wider English population with regard to adult obesity and several behavioural characteristics, such as physical activity levels and smoking^(^ ^45^ ^)^.

A range of measures of SES were used, which facilitated exploration of potential relationships between socio-economic disadvantage and consumption of main meals from different sources. This is particularly important, given evidence that different indicators of SES are associated with different facets of home cooking^(^ ^7^ ^,^ ^29^ ^,^ ^32^ ^,^ ^46^ ^)^. Participants also provided detailed meal consumption data, which enabled a broad understanding of the construction of their diets. Many dietary studies to date have been limited to information on specific nutritional components, such as food items collected through an FFQ, or have studied food preparation or purchasing practices^(^ ^25^ ^,^ ^28^ ^)^. Individuals may prepare or purchase food without eating it themselves and may consume foods they have not themselves prepared or purchased, and such foods may be prepared inside or outside the home. Therefore, focusing on meal consumption as here is likely to offer a more accurate measure of exposure. Previous work has often concentrated solely on binary in-home *v.* out-of-home food intake; however, given the ambiguity of terminology around meals cooked at home and obtained from alternative sources, there is often no clear distinction for location of preparation and consumption^(^ ^25^ ^,^ ^33^ ^)^.

This research is also subject to limitations. In 2011, the median age of the UK population was 39 years^(^ ^47^ ^)^; however, the median age of Fenland participants included in analyses was 48·9 years. Participants were aged 29–64 years at recruitment and were therefore not representative of the full UK population age distribution. In 2011, 86·0 % of the UK population identified themselves as ‘white’^(^ ^47^ ^)^, compared with 97·3 % of the sample included in our analyses. The proportion of participants included in our analyses who reported working in the past four weeks was 87·7 %, whereas in 2011 the employment rate for 16–64-year-olds in the wider UK population was 71·0 %^(^ ^47^ ^)^. The participant sample excluded people with previously diagnosed diabetes, which could have affected interpretation of the potential association between consumption of meals from different sources and development of non-communicable diseases. We also excluded people with missing data on any variable described in the analysis, and the excluded participants differed systematically from the rest of the cohort in terms of some characteristics (see Table 1). It is possible therefore that our results may not necessarily be generalisable to the wider UK population; however, less than 10 % of the original sample was excluded.

Our study lacked details of household composition, which has previously been identified as important in influencing food preparation patterns, particularly among employed parents^(^ ^32^ ^,^ ^48^ ^)^, and meant our measure of household income was not equivalised for household composition. Data on the number of persons and number of children in the household, gender and marital relationships would have enabled additional interpretation of the findings, and more specific recommendations regarding population groups that cook at home infrequently. Study participants self-reported sociodemographic characteristics and meal consumption patterns. In common with similar studies on frequency of consuming different meal types^(^ ^25^ ^,^ ^28^ ^)^, the specific questionnaire items were not validated and may therefore have been interpreted differently by different people. Given the general lack of clarity and variability in definitions of meal sourcing, particularly regarding ‘home cooking’ and ‘eating out’, this highlights the need for improved terminology, conceptualisation and operationalisation in dietary studies.

Participants may have under-reported consumption of ready meals and takeaways if they perceived these to be unhealthy and therefore less socially desirable. If this bias differed by socio-economic group, it could obscure true associations between SES and meal consumption patterns. Since there were differences between participants included and excluded from our analyses, the results may represent upper-bound estimates, if participants responding to the study questionnaire consumed healthier diets and/or had greater nutritional knowledge and awareness than non-responders. Previous studies have undertaken categorisation of related behaviours, for example in terms of cooking frequency (0–1, 2–5 or 6–7 times per week)^(^ ^13^ ^,^ ^32^ ^)^ and frequency of consuming midday or evening meals prepared at home (0–2, 3–4 or 5–7 times per week)^(^ ^18^ ^)^. We collapsed frequency of meal consumption into binary categories, to enable statistical analyses and clear comparisons between main meals from different sources, which exhibited different frequency distributions. However, this may have inhibited some interpretation of nuances around meal consumption patterns. Participants who were not currently working were allocated to the ‘not working overtime’ group, which could have obscured some of the detail regarding the relationship between overtime working and patterns of meal consumption. Although our analyses adjusted for several relevant potential confounding factors, residual confounding is always possible.

### Interpretation of findings

Overall, the patterning of meal sourcing behaviour by sociodemographic factors identified in the present study indicated the presence of embedded cultural norms. Some of these are likely to be generational and influenced by the prevailing cultural context, and hence may be expected to change over time. Existing evidence from systematic reviews suggests that preparing food at home and eating home-cooked meals are likely to provide benefits to diet and health over obtaining meals from other sources^(^ ^25^ ^,^ ^28^ ^)^. Therefore, public health strategies to improve diet and health may focus on increasing consumption of home-cooked meals; decreasing consumption of alternative meal types; and/or improving the healthiness of meals from other sources. This research provides important insights regarding the most effective targeting of interventions to shift patterns of meal consumption towards healthier practices.

In the present study we identified an association between being female and eating home-cooked meals more frequently. This concurs with results^(^ ^13^ ^)^ from the US National Health and Nutrition Examination Survey (NHANES)^(^ ^49^ ^)^. In contrast, analysis of data from the UK National Diet and Nutrition Survey (NDNS)^(^ ^50^ ^)^ found that similar proportions of men and women lived in households where the main food provider (defined as the person in the household with the main responsibility for shopping and preparing food) prepared a main meal on most days of the week^(^ ^51^ ^)^. However, women were more likely than men to prepare meals themselves on at least five days of the week, and the NDNS analysis focused on food preparation rather than consumption, hence interpretation is reliant on the assumption that meal availability is associated with subsequent intake. Furthermore, participation in the NDNS and NHANES may be biased by the substantial commitment involved in taking part, which could affect resultant findings^(^ ^50^ ^)^. Our research identified that being male was associated with a higher frequency of consumption for all out-of-home main meal types. Similarly, previous research has shown that men purchased more out-of-home meals than women^(^ ^52^ ^)^ and men were more likely to report eating fast food, takeaways and ready meals^(^ ^53^ ^,^ ^54^ ^)^.

We found a small association between older age and more frequent consumption of home-cooked meals. This is in agreement with a study of US health professionals, which identified that those consuming a higher frequency of home-cooked meals were likely to be older^(^ ^18^ ^)^. Similarly, in the NDNS, older participants (50–64 years) were more likely than younger (19–34 years) to live in a household where the main food provider prepared a main meal on most days of the week, although the relationship with age was non-linear^(^ ^51^ ^)^. Given the associations between frequency of consuming home-cooked meals, age and gender, there may be a generational effect in meal sourcing, such that older women are likely to eat home-cooked meals more frequently due to historical societal expectations and priorities.

More frequent consumption of home-cooked meals was associated with not working overtime, whereas a higher frequency of eating meals out was associated with overtime working. This indicates, in accordance with previous research^(^ ^2^ ^,^ ^55^ ^–^ ^57^ ^)^, that lack of time, including time constraints due to employment, may be a potential barrier to eating home-cooked food. Policy makers may therefore focus on promoting time-efficient cooking approaches, and development of time-saving skills such as batch cooking, through classes to develop wider food skills beyond those directly related to technical cooking tasks. Policies addressing working patterns, to reduce overtime working, could also offer benefits.

In the present study, higher SES in terms of educational attainment and household income was associated with a higher frequency of eating home-cooked meals and meals out, and a lower frequency of consuming takeaways. This suggests that health promotion messages regarding the potential negative implications of takeaways for diet and health may have been differentially adopted according to SES, which could lead to widening of diet-related health inequalities.

However, relationships between SES and meal consumption were not consistent across all measures of SES. The associations between meal consumption frequency and occupational status, and employment status, were no longer significant after adjustment for other sociodemographic variables. This variation could be because different SES indicators may reflect different aspects of socio-economic position, and hence using measures together helps to avoid the potentially spurious associations identified when using measures separately.

In general, associations were observed only for the highest compared with the lowest category of variables, and not for the intermediate compared with the lowest category of variables. Although trends within categories were not tested statistically here, exploration using greater granularity within measures of SES could provide an opportunity for future research. The relationship between educational attainment and meal consumption may indicate that education itself, rather than its use as a measure of SES, is important in determining meal sourcing behaviour. For example, education might help develop problem-solving skills, enabling people to overcome barriers in order to cook at home. Higher educational attainment could also indicate greater potential exposure to cooking skills training in an educational setting, and greater development of nutritional knowledge and both health literacy and food literacy^(^ ^58^ ^)^. For ready meals, the only significant association with meal consumption frequency in terms of SES was for the highest compared with the lowest income category, which showed less frequent ready meal consumption. Overall, these relationships may indicate that in lower SES strata, with potentially fewer resources, takeaways may be perceived as a more cost-effective or attractive alternative to cooking at home and eating out than ready meals.

Data from the NDNS previously showed that adults of higher SES, as measured by occupational status and age at completion of full-time education, were more likely to eat out at least once per week, although there was no observed association between SES and takeaway consumption^(^ ^33^ ^)^. In a systematic review, higher SES was overall associated with higher dietary energy derived from eating out of home, defined as including both place of consumption and place of preparation of food^(^ ^25^ ^)^. This was particularly evident when measuring SES using higher household income^(^ ^59^ ^,^ ^60^ ^)^ and higher educational attainment^(^ ^61^ ^–^ ^63^ ^)^. It is likely that at least some of the discrepancies between different studies may be attributable to varying terminology regarding main meal alternatives to home-cooked meals and nuances between different measures of SES. Disparities between studies may also be due to cultural variation in terms of leisure pursuits^(^ ^64^ ^)^, cooking practices^(^ ^65^ ^)^ and value placed on cooking^(^ ^66^ ^)^.

The association between higher SES and higher frequency of eating home-cooked meals observed in our research may indicate links between socio-economic disadvantage and fewer resources, kitchen facilities and/or skills for cooking meals at home^(^ ^67^ ^,^ ^68^ ^)^. It is also possible that home-cooked meals may be more highly valued culturally among SES groups with higher levels of educational attainment, or that cooking at home is equally valued across the socio-economic spectrum, but those of higher household income have greater resources and financial opportunity to engage in cooking. The relationship between frequency of consuming home-cooked meals and SES may be influenced by food price, given that cooking involves the use of basic ingredients such as fruit and vegetables. The association between dietary costs and fruit and vegetable intake is stronger for lower-income and less-educated groups, suggesting actual barriers to purchasing ingredients^(^ ^38^ ^)^, and perceptions of healthy food availability are also known to be important in determining behaviour^(^ ^69^ ^,^ ^70^ ^)^. Public health interventions to promote home cooking may therefore need to be more targeted at lower-SES groups and supported by measures to increase affordability of basic ingredients.

### Unanswered questions and future research

To establish causal relationships, longitudinal studies investigating associations between sociodemographic characteristics and patterns of consuming home-cooked main meals and meals from out-of-home sources are required. To achieve this, questions on meal sourcing and consumption could be embedded into existing national longitudinal studies, and future longitudinal analysis will also be possible through phase two follow-up data collection in the Fenland Study. Additionally, regular surveys are needed to identify secular trends in meal sourcing and consumption, such that public health initiatives may be tailored to prevailing and prospective patterns of behaviour. More novel approaches might include utilising existing data sources, such as exploring associations between sociodemographic characteristics and retail data in supermarket loyalty programmes.

In future it will be important to address nuances around different measures of SES and potential associations with main meal patterns, for example exploring whether relationships differ according to education or wealth. Development of more objective measures of leisure time availability and employment patterns, and their relationship with meal sourcing, would also prove insightful. The current study did not comprise an ethnically diverse sample, and investigation of meal patterns among people from different ethnic and cultural backgrounds would help to further understanding of these relationships. Identifying the relative contributions of home-cooked meals and different out-of-home meal types to individuals’ overall diets, using both quantitative and qualitative data, would provide additional insights to help guide public health policies and interventions encouraging healthier dietary patterns. Finally, clear, consistent terminology around home-cooked meals, convenience foods, eating out and food from other sources needs to be developed, to enable informed comparisons and conclusions in research and effective public health promotion.

## Conclusions

In a population-based cross-sectional study, the sociodemographic characteristics associated with frequency of eating meals from different out-of-home sources varied according to meal source. A higher frequency of eating home-cooked meals was associated with being female, older, not working overtime and higher SES (measured by greater educational attainment and household income). Consuming takeaways more frequently was associated with lower SES (measured by lower educational attainment and household income) and consuming ready meals more frequently was associated with lower SES (measured by household income only). Eating meals out more frequently was associated with being male, working overtime and higher SES (measured by greater educational attainment and household income). These findings may be used to help targeting of public health policies and interventions promoting healthier diets and dietary-related health towards specific population groups, such people working overtime, those of lower educational attainment and household income, younger individuals and men. Further research is required to: establish causal relationships between sociodemographic characteristics and meal sourcing; determine how to change patterns of consumption behaviour most effectively; and evaluate potential associations between dietary intakes and patterns of meal sourcing.
